# Classroom Size and the Prevalence of Bullying and Victimization: Testing Three Explanations for the Negative Association

**DOI:** 10.3389/fpsyg.2019.02125

**Published:** 2019-09-20

**Authors:** Claire F. Garandeau, Takuya Yanagida, Marjolijn M. Vermande, Dagmar Strohmeier, Christina Salmivalli

**Affiliations:** ^1^Department of Psychology, University of Turku, Turku, Finland; ^2^Department of Applied Psychology, Work, Education and Economy, University of Vienna, Vienna, Austria; ^3^Department of Child and Adolescent Studies, Utrecht University, Utrecht, Netherlands; ^4^Department of Social Work, School of Medical Engineering and Applied Social Sciences, University of Applied Sciences Upper Austria, Linz, Austria; ^5^Department of Psychology, Shandong Normal University, Jinan, China

**Keywords:** bullying, victimization, class size, aggression, multilevel analyses

## Abstract

Classroom size - i.e., the number of students in the class - is a feature of the classroom environment often found to be negatively related to bullying or victimization. This study examines three possible explanations for this negative association: (a) it is due to measurement effects and therefore only found for peer-reports (Hypothesis 1), (b) bullying perpetrators are more popular and have more friends in smaller classrooms (Hypothesis 2), (c) targets of bullying are more popular and have more friends in larger classrooms (Hypothesis 3). Multilevel regression analyses were conducted on a sample from Austria (1,451 students; *M*age = 12.31; 77 classes) and a sample from the Netherlands (1,460 students; *M*age = 11.06; 59 classes). Results showed that classroom size was negatively associated with peer-reported bullying and victimization in both samples, and with self-reported bullying and victimization in the Dutch sample only, suggesting partial support for Hypothesis 1. Students high in bullying were found to be more popular in smaller than in larger classrooms in the Austrian sample. The negative link between victimization and popularity was found to be stronger in smaller classrooms than in larger classrooms in the Dutch sample. However, classroom size was not found to moderate links between bullying or victimization and friendship in either sample. Hypotheses 2 and 3 were supported, but only for popularity and in a single sample. Further research is needed to better understand the higher prevalence of bullying found in smaller classrooms in many studies.

## Introduction

The prevalence of bullying and victimization in classrooms is not merely the result of individual characteristics of the bullying perpetrators and their targets but is influenced by features of the classroom environment (Saarento et al., 2015). These contextual characteristics include the anti-bullying attitudes and behaviors of peer bystanders (Salmivalli et al., 2011) and of teachers (Veenstra et al., 2014; Oldenburg et al., 2015), as well as aspects of the peer social network, such as the degree of status hierarchy in the classroom (Garandeau et al., 2014). Classroom size - i.e., the number of students in the class - is a structural feature that has often been investigated in relation to academic achievement (see Finn et al., 2003), with smaller classrooms often found to be beneficial for academic performance (Hoxby, 2000; Shin and Raudenbush, 2011) and even earnings later in life (Fredriksson et al., 2013). Intuitively, we would expect the same advantageous effects of small classrooms on bullying. Smaller classrooms should logically protect against bullying thanks to higher adult/child ratios, allowing a more effective monitoring of children’s negative behaviors by school personnel.

Classroom size has been investigated in many studies on victimization and bullying, often as a control variable rather than a main predictor of interest. Surprisingly, very few studies found evidence of a protective effect of smaller classroom networks on bullying or victimization (Whitney and Smith, 1993; Khoury-Kassabri et al., 2004). The large majority of studies examining the link between classroom size and bullying or victimization found them to be either negatively associated (e.g., Vervoort et al., 2010) or unrelated (e.g., Thornberg et al., 2017). However, the reason why bullying and victimization would be more prevalent in smaller classrooms remains unclear.

The present study aims to test for three possible explanations for this negative association: First, the negative association may not reflect an actual social phenomenon but result from a measurement effect, related to the way peer-reported scores are computed. In this case, the prevalence-size link should be negative for peer-reported, but not for self-reported bullying and victimization (Hypothesis 1). Second, it is possible that bullying perpetrators enjoy higher status and are more socially connected in smaller classrooms, which in turn facilitates their bullying behavior. Engaging in bullying may be associated with higher perceived popularity and more friendships in smaller than in larger classrooms (Hypothesis 2). Third, victims may have less social support and fewer opportunities for friendships in smaller classrooms, which in turn could contribute to the maintenance of their victimization. Being victimized may be associated with lower perceived popularity and fewer friendships in smaller than in larger classrooms (Hypothesis 3). These hypotheses will be tested with large samples from two countries, using both self-reports and peer-reports of bullying and victimization.

### Associations of Classroom Size With Bullying and Victimization: Does Informant Type Matter?

Contrary to expectations, research seldom found support for a positive link between classroom size and bullying and victimization (Whitney and Smith, 1993; Khoury-Kassabri et al., 2004). It is noteworthy that these studies used only self-reports and did not operationalize class size as the exact number of students in each classroom but as the average class size in the schools (by dividing the number of students in the school by the number of classrooms). In the review of the literature, the type of informant - self or peers – appears to be relevant for the strength and direction of the association between classroom size and bullying or victimization. All studies showing no significant association between these variables used self-reported measures of bullying (O’ Moore et al., 1997; Boyesen and Bru, 1999; Wolke et al., 2001; Scheithauer et al., 2006; Salmivalli et al., 2011; Coehlo and Sousa, 2018) or victimization (Whitney and Smith, 1993; O’ Moore et al., 1997; Wolke et al., 2001, German sample; Stefanek et al., 2011; Saarento et al., 2013; Thornberg et al., 2017; Coehlo and Sousa, 2018).

Negative associations between classroom size and bullying or victimization have been found with both self-reported and peer-reported measures. A higher classroom size was found to be associated with a lower prevalence of peer-reported bullying (Vervoort et al., 2010; Garandeau et al., 2014) peer-reported victimization (Vervoort et al., 2010; Saarento et al., 2013), self-reported bullying (Stefanek et al., 2011) and self-reported victimization (Wolke et al., 2001, British sample; Verkuyten and Thijs, 2002). Furthermore, a study using dyadic nominations of who bullies whom showed that there was less bullying in classrooms with a higher number of students (Tolsma et al., 2013). When measures of effect size were available for these studies, which were heterogeneous in various methodological aspects (e.g., sample, demographics, control variables etc.), they indicated that each additional student in the classroom was associated with a decrease of 0.06 to 0.1 (on a scale of 0 to 1) in peer-reported measures and of approximately 0.02 (on a scale of 0 to 4) in self-reported measures.

Taken together, these findings hint that the negative link between classroom size and bullying or victimization may be partly accounted for by the measurement of the variables. It would hold mainly for peer-reported bullying and victimization - which are obtained by computing proportions of nominations received by peers. It is possible indeed that, in small classrooms, the probability is higher than in large classrooms for students to score high in peer-reported measures, since they are computed by dividing the number of nominations received by classmates by the number of nominators. As smaller classrooms have fewer nominators, students receiving for example only one or two nominations should score higher in smaller than in larger classrooms. As self-reports rely on single informants, the number of participants in the classroom should not affect self-reported scores.

### Moderating Effects of Classroom Size on the Social Power of Perpetrators and Targets?

Another explanation for findings of a lower prevalence of bullying in larger classrooms is that bullying perpetrators, who tend to be perceived as more popular than non-bullying students (e.g., Caravita et al., 2009), may have even more social power in smaller classrooms. As bullying often involves the manipulation of a peer group by one or two bullies (see Garandeau and Cillessen, 2006), it might be more difficult for children who initiate bullying to exert influence over the whole peer network when this network is large. Opposing the ring-leader bullies might be more challenging in more restricted social environments. Similarly, bystanders may be more likely to side with the bullies in smaller classes. This support for bullying perpetrators could be reflected in higher levels of popularity, an indicator of influence among peers, and a higher number of friends for bullies in smaller classrooms. In turn, those who engage in bullying behavior should be more apt to maintain and even increase such behavior if they are socially rewarded for it. Therefore, the negative association between classroom size and bullying (or victimization) could be explained by the higher levels of perceived popularity and the higher number of friends for bullying perpetrators in smaller classrooms relative to larger classrooms.

A third explanation is the possibility that victims may have less social power or support in smaller classrooms. Victimized children generally have low levels of popularity (e.g., Pouwels et al., 2016) and are less likely than other children to have reciprocal friends (e.g., Scholte et al., 2009). For these victimized children, it may be more difficult to find at least one friend in smaller compared to larger classrooms, as the number of possible social connections is more limited. This restriction in friendship opportunities for victims could account for higher victimization levels in smaller classrooms, as having friends tends to protect against victimization (Boulton et al., 1999; Hodges et al., 1999; Serdiouk et al., 2016).

### The Present Study

The relation of class size to bullying and victimization needs to be better understood. The two main objectives of the present study were a) to test for the direction (positive or negative) of the association of classroom size with bullying and victimization, using both peer-reports and self-reports, in two samples from two countries; b) to put to the test three possible explanations for these surprising findings.

First, we formulated the general hypothesis that the effect of classroom size on peer-reported bullying and peer-reported victimization would be negative, on the basis of previous findings from the literature. To further explore this effect, we tested for both linear and curvilinear associations. The first explanation that we put to the test was that this negative association was due to a measurement effect. Negative links would be found with peer-reports of bullying and victimization, but not with self-reported measures (Hypothesis 1). Our second hypothesis was that bullying perpetrators enjoyed higher social power in smaller classrooms. If this holds true, the associations between bullying and perceived popularity, and between bullying and having friends, would be stronger in smaller classrooms (Hypothesis 2). Our third hypothesis was that victimized children had less social power in smaller classrooms. If this holds true, children higher in victimization would have lower perceived popularity and fewer friends in smaller classrooms (Hypothesis 3).

We tested these hypotheses with participants in late childhood and early adolescence, as this is the age when school bullying problems tend to be the most prevalent. To provide a more valid test of our hypotheses, we chose to conduct our analyses on two datasets from two countries, Austria and the Netherlands. The objective of the present study was not to compare findings between the two samples or countries, but rather to increase the generalizability of the results. The Austrian sample was previously used in the study by Stefanek et al. (2011), which did examine classroom size in relation only to self-reported victimization and bullying.

## Materials and Methods

### Samples and Procedures

Similar - though not identical - datasets were available for the two samples. The Austrian sample included 1,451 fifth- to eighth-graders (mean age: 12.31, *SD* = 1.20; 51.2% boys) from 77 classrooms in 11 schools. Classroom size ranged from 8 to 28 students (*M* = 18.84; *SD* = 4.39). The majority of participants were born in Austria (53.2%). Before a student could participate in the study, an active and written consent was required from parents, who had to sign and return an informed consent form. Only the students with an active, written and informed consent from their parents were invited to participate in the study. Moreover, participation in the study was voluntary and strictly confidential, resulting in a participation rate of 90%. Data was collected in 2008, in the middle of the school year. The questionnaires were completed during regular teaching hours in the schools’ computer labs, under the supervision of two trained research assistants. It is the pre-test data of the ViSC Austrian program, which was designed to increase social competence in school children. In Austria, an ethics approval is mandatory and required. The procedure took place in three steps: First, the study was approved by the ethics committee located in the Austrian Ministry of Education; second, it was approved by the ethics committee located in the Federal School Directorate; third, it was approved by each individual school leader (rector). An ethics approval from the authors’ Institutions’ Ethics Committee was not required as per applicable institutional and national guidelines and regulations.

The Dutch sample consisted of 1,460 children and early adolescents (49,8% boys) from 59 classrooms in 40 schools. Classroom size ranged from 15 to 33 (*M* = 24.75, *SD* = 4.10). The students were in grades 4 to 6, which are the last 3 years of elementary school in the Dutch school system. Their mean age was 11.07 years (*SD* = 0.99). The large majority (96.2%) was Native Dutch. Data were cross-sectional but collected in the spring of three consecutive years from 2010 to 2012. Written informed consent from parents and assent from the child were required to participate in the study. Parents received a letter in which the purposes and procedures of the study were explained. They could refuse participation by returning a pre-printed objection against their child’s participation in the study. This passive consent procedure was in line with the local ethical guidelines at the time of the data collection. When informing adolescents about the aims and procedures of the study, they could choose not to participate (active consent), but no one did so. The participation rate was 98.3%. Self-reports of bullying and victimization were completed during group testing sessions run by trained research assistants. A clinician was made available in case a child would be troubled by the data collection, but that was never the case. The peer nomination questionnaires were administered individually by a research assistant in an interview session in the school of the participating children. The interviewers used laptop computers with a precise protocol to ensure that the questions were administered correctly and consistently.

The data was collected by researchers from the Faculty of Social and Behavioral Sciences at Utrecht University. The Faculty Ethics Review Board (FERB) assumes that all the research at the Faculty of Social and Behavioral Sciences is conducted in an ethically responsible manner in accordance with the prevailing conduct and professional codes and (European, national and international) legislation. No approval was sought from the FERB, as the researchers followed the ethical guidelines of that time and therefore deemed explicit approval not necessary. All schools participating in the study approved of the procedure beforehand. An ethics approval from the authors’ Institutions’ Ethics Committee was not required as per applicable institutional and national guidelines and regulations.

### Measures

#### Self-Reported Bullying

In each sample, self-reported bullying was assessed with one global item. In the Austrian sample, this item was *How often have you insulted or hurt other students during the last 2 months?* In the Dutch sample, the item was from the Olweus questionnaire (Olweus, 1996): *How often have you bullied others at school during the last couple of months?* Responses were given on a five-point scale (ranging from 0 = never to 4 = several times a week).

#### Self-Reported Victimization

Self-reported victimization was also assessed with a single global item. In the Austrian sample, the question was *How often have other students insulted or hurt you during the last 2 months?* In the Dutch sample, the item was *How often have you been bullied at school during the last couple of months?* (Olweus, 1996). The response scales were the same as for self-reported bullying.

#### Peer-Reported Bullying

In both samples, within-classroom peer nominations were obtained by asking participants to provide the names (or check the name from a roster) of classmates enacting the behavior. For each participant, a proportion score was computed by dividing the number of nominations received by the number of nominators. In the Austrian sample, a single question (for global aggression) was used, and nominations originated from victims rather than all participating classmates, based on the approach of Veenstra et al. (2010). Only children who had self-identified as victims in the self-assessments (i.e., children who did not respond “never” to the question *How often have other students insulted or hurt you during the last 2 months?*) were provided with the additional question “*Who insulted or hurt you during the last 2 months*?” and could nominate who the perpetrators were. They could choose up to five classmates. They could also refuse to answer or state that the perpetrator was not a classmate. Therefore, peer-reported bullying in that sample corresponded to the proportion of nominations received as perpetrators of bullying by victims (see Gradinger et al., 2012). No definition of bullying was provided to the participants. In the Dutch sample, perpetration of bullying was assessed with the Bullying Role Nomination Procedure (BRNP; Olthof et al., 2011), which is an adaptation of the Participant Role Questionnaire (Salmivalli et al., 1996). Participants were first instructed that bullying involved (1) intent to harm, (2) repetition over time, (3) power differential, and (4) could take different forms. Five types of bullying were assessed using one peer nomination item for each type: physical, verbal, material (e.g., stealing or destroying things that belong to others), direct relational (e.g., ignoring), indirect relational (e.g., saying nasty things about someone to damage their reputation). The proportion scores were averaged across the five items (α = 0.89).

#### Peer-Reported Victimization

The assessment of peer-reported victimization was analogous to peer-reported bullying, i.e., based on within-classroom peer nominations and the computation of proportion scores. In the Austrian sample, the single question for peer-reported victimization corresponded to the proportion of nominations received as victims of bullying by nominators who had self-identified as perpetrators of bullying in the self-assessments. Only those who had self-identified as perpetrators were asked “*Whom did you insult or hurt you during the last 2 months*?”. In the Dutch sample, victimization was assessed with five items tapping into physical, verbal, material, direct relational, and indirect relational victimization (α = 0.89; BRNP; Olthof et al., 2011). The proportion scores were averaged across the five items.

#### Perceived Popularity

A single peer-nominated item – Who are the most popular in your class? - was used to measure perceived popularity in the two samples. Nominations were limited to five classmates in the Austrian sample and unlimited in the Dutch sample. Proportion scores were computed by dividing the number of nominations received by the number of nominators.

#### Friendships

In both samples, a standard sociometric procedure was used to assess friendships. The number of friendships was operationalized as the proportion of nominations received as “best friend”. In the Austrian sample, the adolescents were asked to choose up to three classmates who were their best friends. The item was: *Who are your best friends*? In the Dutch sample, participants were first presented with the following description: *Some children in your class are friends or girlfriends with each other. They like each other very much, they do a lot together, and have a lot of fun. They also help each other and they can work well together.* The description was followed by the question *Which children in your class are your best friends or girlfriends?* The number of nominations was unlimited.

### Analysis Plan

All hypotheses were tested via multilevel modeling. Analyses were conducted in M*plus 7.4.* To test the first hypothesis, the main effects of classroom size on peer-reported bullying, self-reported bullying, peer-reported victimization and self-reported victimization, were examined in a series of four models for each sample. Individual-level predictors age, gender and popularity were controlled for in these models. All predictor variables were grand mean-centered. In addition to the linear effect of classroom size, we examined curvilinear associations between classroom size and bullying and victimization by adding a quadratic term to the models. To avoid coefficients with 3 or more zero digits after the decimal point, the classroom size variable was divided by 10 in these models; thus, each unit increase represents 10 more students instead of one more student. Unstandardized coefficients are presented in Table 2 for bullying and Table 3 for victimization. The standardized effects of classroom size are provided in the text.

To test for the second and third hypotheses, we ran additional models with popularity and having friends as outcomes (see Table 4). In each model, we tested the cross-level interactions of classroom size with bullying and with victimization, to examine whether the levels of popularity and number of friends of students high in bullying and students high in victimization would depend on the size of their classroom. In these models, we could not use both peer-reports and self-reports of bullying and victimization due to multicollinearity issues; we chose to use the self-reports, as their measurement is independent of classroom size (i.e., the number of classmates is not utilized in the computation of the scores in any way, as is the case for peer-reported measures). All predictor variables were grand mean-centered, except for self-reported bullying and victimization. These variables were classroom mean-centered because they were included in cross-level interactions (see Enders and Tofighi, 2007).

In all analyses, we used robust maximum likelihood (MLR) estimators. The intraclass correlations indicated that differences between classrooms in the Austrian sample explained 12.8 and 6.3% of the variance in peer-reported and self-reported bullying, respectively, and 15.2 and 4.2% of the variance in peer-reported and self-reported victimization, respectively. In the Dutch sample, these percentages were 8.1 and 9.2% for peer-reported and self-reported bullying, respectively, and 3.8 and 4.5% for peer-reported and self-reported victimization, respectively.

## Results

### Descriptive Statistics and Correlations

Table 1 shows the descriptive statistics and correlations for the main study variables, at the individual level and at the classroom level. At the individual level, correlations between peer-reports and self-reports were 0.23 and 0.39 for bullying and 0.20 and 0.41 for victimization, in the Austrian and the Dutch sample, respectively, (*p*s = < 0.001). Regarding classroom size, correlations at the classroom level were negative for peer-reported measures of bullying and victimization in both samples, ranging from −0.35 to −0.53 (*p*s = < 0.001). The correlation between class size and self-reported bullying was non-significant in the Austrian sample and negative in the Dutch sample, *r* = −0.38, *p* < 0.01. Correlations between class size and self-reported victimization were non-significant in both samples.

**TABLE 1:** Descriptive statistics and correlations for the main study variables.

	**Austrian**	**Dutch**							
	**M (SD)**	**M (SD)**	**1**	**2**	**3**	**4**	**5**	**6**	**7**
**Individual-level variables**									
1. Age	12.31 (1.20)	11.06 (0.95)	−	0.00	–0.07^∗∗^	0.04	–0.16^∗∗∗^	0.18^∗∗∗^	–0.03
2. Bullying (PR)	0.06 (0.09)	0.05 (0.09)	−0.06^∗^	−	0.05^∗^	0.39^∗∗∗^	0.07^∗∗^	0.46^∗∗∗^	0.02
3. Victimization (PR)	0.04 (0.07)	0.03 (0.07)	0.07^∗∗^	0.27^∗∗∗^	−	0.02	0.41^∗∗∗^	–0.16^∗∗∗^	–0.23^∗∗∗^
4. Bullying (SR)	0.76 (1.01)	0.35 (0.69)	0.09^∗∗∗^	0.23^∗∗∗^	0.13^∗∗∗^	−	0.16^∗∗∗^	0.14^∗∗∗^	0.04
5. Victimization (SR)	0.96 (1.17)	0.74 (1.14)	–0.04	0.05	0.20^∗∗∗^	0.27^∗∗∗^	−	–0.11^∗∗∗^	–0.16^∗∗∗^
6. Popularity	0.10 (0.13)	0.11 (0.19)	–0.01	0.14^∗∗∗^	–0.10^∗∗∗^	0.13^∗∗∗^	–0.05	−	0.27^∗∗∗^
7. Friendship	0.13 (0.09)	0.14 (0.09)	−0.06^∗^	0.02	–0.13^∗∗∗^	0.02	–0.08^∗∗^	0.48^∗∗∗^	−
**Classroom-level variables**									
1. Class size	18.84 (4.39)	24.75 (4.10)	−	–0.53^∗∗∗^	–0.50^∗∗∗^	–0.38^∗∗^	–0.23		
2. Bullying (PR)	0.06 (0.04)	0.05 (0.03)	–0.35^∗∗^	−	0.88^∗∗∗^	0.70^∗∗∗^	0.50^∗∗∗^		
3. Victimization (PR)	0.04 (0.03)	0.04 (0.02)	–0.36^∗∗^	0.60^∗∗∗^	−	0.56^∗∗∗^	0.57^∗∗∗^		
4. Bullying (SR)	0.77 (0.36)	0.37 (0.26)	–0.15	0.26^∗^	0.55^∗∗∗^	−	0.38^∗∗^		
5. Victimization (SR)	0.95 (0.36)	0.75 (0.34)	0.10	0.54^∗∗∗^	0.28^∗^	0.46^∗∗∗^	−		

*Correlations for the Dutch sample are presented above the diagonal; correlations for the Austrian sample are presented below the diagonal. PR, peer-reported; SR, self-reported. The correlations between classroom size and all other classroom-level variables are based on classroom means of the individual-level variables. ^∗^*p* < 0.05, ^∗∗^*p* < 0.01, ^∗∗∗^*p* < 0.001.*

### Main Effects of Classroom Size on Bullying

Results of the multilevel models testing the effects of age, gender, popularity and classroom size on peer-reported and self-reported bullying are shown in Table 2. Across both samples, boys and more popular students were found to be higher in both peer-reported and self-reported bullying. The effect of age varied across samples and types of bullying: In the Austrian sample, older students reported bullying more than their younger counterparts, but did not have higher levels of peer-reported bullying. In the Dutch sample, older students had lower levels of peer-reported bullying than younger students, but there was no significant effect of age on self-reported bullying.

**TABLE 2:** Main effects of age, gender, popularity and classroom size on peer-reported and self-reported bullying for the two samples.

	**Peer-reported bullying**				**Self-reported bullying**			
	**Austrian sample**		**Dutch sample**		**Austrian sample**		**Dutch sample**	
	**Est. (SE)**	***p***	**Est. (SE)**	***p***	**Est. (SE)**	***p***	**Est. (SE)**	***p***
Intercept	0.059 (0.005)	<0.001	0.043 (0.004)	<0.001	0.776 (0.043)	<0.001	0.290 (0.044)	<0.001
**Student-level**								
Age	0.000 (0.003)	0.994	−0.006 (0.003)	0.040	0.065 (0.026)	0.013	0.018 (0.029)	0.531
Gender	0.046 (0.006)	<0.001	0.033 (0.006)	<0.001	0.187 (0.063)	0.003	0.145 (0.041)	<0.001
Popularity	0.070 (0.018)	<0.001	0.201 (0.025)	<0.001	0.967 (0.249)	<0.001	0.454 (0.128)	<0.001
**Class-level**								
Size (linear)	−0.024 (0.009)	0.007	−0.027 (0.007)	<0.001	−0.092 (0.086)	0.286	−0.160 (0.066)	0.016
Size^2^ (quadratic)	−0.001 (0.020)	0.943	0.044 (0.019)	0.022	−0.092 (0.147)	0.531	0.388 (0.207)	0.062
Res. var._within_	0.006 (0.001)	<0.001	0.005 (0.001)	<0.001	0.941 (0.066)	<0.001	0.418 (0.047)	<0.001
Res. var._between_	0.001 (0.000)	<0.001	0.000 (0.000)	0.003	0.055 (0.019)	0.003	0.031 (0.009)	0.001
LL	1580.518		1724.977		−2016.450		−1437.667	

*Unstandardized estimates (and standard errors) shown. All variables are grand-mean centered. Gender was coded as 0:girl, 1:boy. LL, log-likelihood. Res. var, Residual variance.*

The linear effects of classroom size on peer-reported bullying were negative in both the Austrian sample, *γ* = −0.352, *p* = 0.003, and the Dutch sample, *γ* = −0.474, *p* < 0.001, thus supporting our general hypothesis. Regarding the association between classroom size and self-reported bullying, there was no significant effect in the Austrian sample, *γ* = −0.170, *p* = 0.304, but a negative effect was found in the Dutch sample, *γ* = −0.309, *p* = 0.006. Only the results from the Austrian sample were consistent with Hypothesis 1, according to which a negative effect would be observed for peer-reported bullying, but not for self-reported bullying. The proportion of between-classroom variance in peer-reported bullying explained by classroom size (linear effects) was 12% in the Austrian sample and 33.5% in the Dutch sample. For self-reported bullying, classroom size explained 3 and 16.7% of the between-class variance in the Austrian sample and in the Dutch sample, respectively.

In the Austrian sample, there was no evidence of a curvilinear association of classroom size with any of the two outcomes. In the Dutch sample, however, there was a significant positive quadratic effect for peer-reported bullying, *p* = 0.022, suggesting that peer-reported bullying decreases until class size reaches approximately 29 students and starts increasing when the number of students in the classroom is 30 (the maximum being 33).

### Main Effects of Classroom Size on Victimization

The results are presented in Table 3. There was no significant effect of age, except for younger Dutch students being more likely than older ones to report being victimized. Boys had higher levels of peer-reported victimization in the Austrian sample only. More popular students were less likely to be perceived as victimized by peers in both samples, in the Austrian sample.

**TABLE 3:** Main effects of age, gender, popularity and classroom size on peer-reported and self-reported victimization for the two samples.

	**Peer-reported victimization**				**Self-reported victimization**			
	**Austrian sample**		**Dutch sample**		**Austrian sample**		**Dutch sample**	
	**Est. (SE)**	***p***	**Est. (SE)**	***p***	**Est. (SE)**	***p***	**Est. (SE)**	***p***
Intercept	0.037 (0.004)	<0.001	0.030 (0.003)	<0.001	0.987 (0.049)	<0.001	0.688 (0.048)	<0.001
**Student-level**								
Age	0.001 (0.002)	0.653	−0.003 (0.002)	0.105	−0.045 (0.034)	0.193	−0.169 (0.039)	<0.001
Gender	0.011 (0.005)	0.018	0.003 (0.004)	0.451	−0.141 (0.073)	0.052	−0.081 (0.052)	0.122
Popularity	−0.069 (0.011)	<0.001	−0.061 (0.007)	<0.001	−0.400 (0.219)	0.068	−0.557 (0.137)	<0.001
**Class-level**								
Size (linear)	−0.022 (0.008)	0.008	−0.023 (0.001)	<0.001	0.029 (0.095)	0.761	−0.178 (0.080)	0.026
Size^2^ (quadratic)	0.026 (0.022)	0.246	0.029 (0.009)	0.001	−0.147 (0.151)	0.329	0.329 (0.166)	0.047
Res. var._within_	0.004 (0.000)	<0.001	0.004 (0.001)	<0.001	1.298 (0.066)	<0.001	1.211 (0.077)	<0.001
Res. var._between_	0.001 (0.000)	0.007	0.000 (0.000)	0.214	0.055 (0.019)	0.003	0.026 (0.019)	0.162
LL	1949.283		1837.381		−2254.944		−2183.182	

*Unstandardized estimates (and standard errors) shown. All variables are grand-mean centered. Gender was coded as 0:girl, 1:boy. LL, log-likelihood. Res. var, residual variance.*

Consistent with our general hypothesis, the linear effects of classroom size on peer-reported victimization were negative in both the Austrian sample, *γ* = −0.341, *p* = 0.001, and the Dutch sample, *γ* = −0.596, *p* < 0.001. In line with our findings for bullying, classroom size was not significantly associated with self-reported victimization in the Austrian sample, *γ* = −0.052, *p* = 0.761, but this association was significantly negative in the Dutch sample, *γ* = −0.367, *p* = 0.032. Thus, support was found for Hypothesis 1 in the Austrian sample, but not in the Dutch sample. The proportion of between-classroom variance in peer-reported victimization explained by classroom size (linear effects) was 19% in the Austrian sample and 46.3% in the Dutch sample. The proportion of between-classroom variance in self-reported victimization explained by classroom size was 0.3% in the Austrian sample and 16.7% in the Dutch sample.

Consistent with our findings for bullying, there was no evidence of a curvilinear association of classroom size with either self- or peer-reported victimization in the Austrian sample. In the Dutch sample, there was a significant positive quadratic effect for peer-reported victimization, *p* = 0.001, and self-reported victimization, *p* = 0.047. For both measures, victimization decreases until class size reaches 29 students and starts increasing again when classrooms include at least 30 students.

### Interactive Effects of Classroom-Size on Popularity

The models testing whether the associations of bullying and victimization with popularity were moderated by classroom size are shown in Table 4. There was no significant effect of gender on popularity in either sample. Older students tended to be more popular in the Dutch sample only. In both samples, students higher in bullying were more popular and those higher in victimization were less popular.

**TABLE 4:** Main and interactive effects of age, gender, bullying, victimization, classroom size on popularity and friendship for the two samples.

	**Popularity**				**Friendship**			
	**Austrian sample**		**Dutch sample**		**Austrian sample**		**Dutch sample**	
	**Est. (SE)**	***p***	**Est. (SE)**	***p***	**Est. (SE)**	***p***	**Est. (SE)**	***p***
Intercept	0.150 (0.005)	<0.001	0.117 (0.004)	<0.001	0.136 (0.002)	<0.001	0.149 (0.003)	<0.001
**Student-level**								
Age (years)	0.000 (0.004)	0.937	0.031 (0.016)	0.049	−0.003 (0.002)	0.221	−0.004 (0.002)	0.081
Gender	0.000 (0.006)	0.937	0.012 (0.008)	0.145	−0.005 (0.005)	0.314	−0.002 (0.004)	0.640
Bullying (SR)	0.026 (0.006)	<0.001	0.044 (0.012)	<0.001	0.004 (0.004)	0.308	0.000 (0.004)	0.923
Victimization (SR)	−0.011 (0.003)	0.001	−0.022 (0.004)	<0.001	−0.008 (0.002)	<0.001	−0.017 (0.002)	<0.001
**Classroom-level**								
Size	−0.004 (0.001)	0.009	−0.003 (0.001)	0.001	−0.005 (0.001)	<0.001	−0.007 (0.001)	<0.001
**Cross-level interactions**								
Size^∗^bullying	−0.003 (0.001)	0.028	−0.001 (0.002)	0.576	−0.001 (0.001)	0.415	0.000 (0.001)	0.702
Size^∗^victimization	0.001 (0.001)	0.372	0.002 (0.001)	0.035	0.001 (0.000)	0.273	0.001(0.001)	0.175
Res. variance_within_	0.017 (0.001)	<0.001	0.032 (0.002)	<0.001	0.008 (0.001)	<0.001	0.007 (0.000)	<0.001
Res. variance_between_	0.000 (0.002)	0.996	0.000 (0.002)	0.995	0.000 (0.000)	0.994	0.000 (0.000)	0.262
Res. variance_slope–bullying_	0.000 (0.000)	0.861	0.003 (0.001)	0.076	0.000 (0.000)	0.962	0.000 (0.000)	0.803
Res. variance_slope–victimization_	0.001 (0.000)	0.087	0.000 (0.000)	0.926	0.000 (0.000)	0.043	0.000 (0.000)	0.896
LL	884.617		418.908		1454.529		1483.043	

*Unstandardized estimates (and standard errors) shown. Age, gender and classroom size are grand mean-centered; Self-reported bullying and victimization are classroom-mean centered. SR, self-reported; LL, log-likelihood; Res. variance, residual variance.*

**FIGURE 1:**
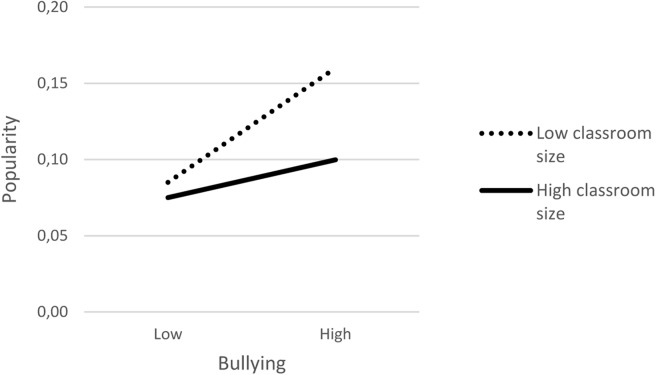
Moderating effects of classroom size on the association between classroom mean-centered self-reported bullying and popularity in the Austrian sample. Cut-offs of 1SD above and below the mean were used to represent the level of popularity of adolescents low and high in self-reported bullying, in small classrooms (∼ 14 students) and in large classrooms (∼ 23 students).

The cross-level interaction between classroom size and bullying was significant in the Austrian sample, suggesting that the association between bullying and popularity does differ depending on the number of students in the class. This significant interaction was probed by plotting the effects of bullying on popularity at high and low levels of classroom size (see Figure 1). The significance of these effects was determined in a simple slope analysis, using the tools provided by Preacher et al. (2006), and choosing +1SD and −1SD as indicators of low and high levels of bullying and classroom size. The slopes of the effects of bullying on popularity were significant and positive both for smaller and larger classrooms, but the slope was stronger for smaller, *b* = 0.039, SE = 0.011, *p* = < 0.001, than for larger classrooms, *b* = 0.013, SE = 0.006, *p* = 0.020. For students with higher levels of bullying, levels of popularity were lower in larger than in smaller classrooms, *b* = −0.007, SE = 0.003, *p* = 0.006, whereas the popularity levels of students with lower levels of bullying did not significantly vary depending on classroom size, *b* = −0.001, SE = 0.001, *p* = 0.364. This finding is consistent with Hypothesis 3. However, the interaction between classroom size and bullying was not significant in the Dutch sample.

**FIGURE 2:**
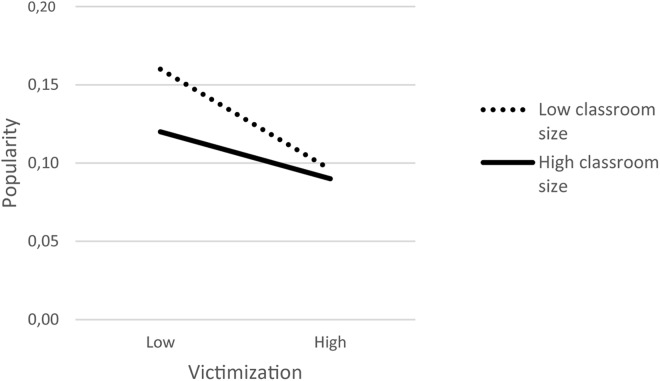
Moderating effects of classroom size on the association between classroom mean-centered self-reported victimization and popularity in the Dutch sample. Cut-offs of 1SD above and below the mean were used to represent the level of popularity of adolescents high and low in victimization, in small classrooms (∼ 21 students) and in large classrooms (∼ 29 students).

The association between victimization and popularity was found to differ depending on classroom size in the Dutch sample only. A graphical representation of the cross-level interaction is shown in Figure 2. The slopes of the effects of victimization on popularity were significant and negative in both smaller and larger classrooms, but the slope was stronger in smaller classrooms, *b* = −0.030, SE = 0.005, *p* = < 0.001, than in larger classrooms, *b* = −0.014, SE = 0.006, *p* = 0.028. Importantly, classroom size had an effect on the popularity levels of students with lower levels of victimization, *b* = −0.005, SE = 0.002, *p* = < 0.001, who were less popular in smaller compared to larger classrooms; however, the popularity levels of students higher in victimization did not significantly vary as a function of classroom size, *b* = 0.001, SE = 0.002, *p* = 0.594. This finding is consistent with Hypothesis 3 to the extent that the negative link between victimization and popularity was stronger in smaller classrooms. Nevertheless, our results suggest that classroom size made a difference only for the popularity of students low in victimization.

### Interactive Effects of Classroom-Size on Having Friends

The models testing for the moderating effects of classroom size on the relations of bullying and victimization with friendship are also shown in Table 4. In both samples, there was no significant effect of age or gender on friendships. There was no indication that classroom size moderated the effects of either bullying or victimization on having friends, in either sample. Therefore, our analyses with friendships did not support either Hypothesis 2 or Hypothesis 3.

## Discussion

The main goal of the present study was to investigate the link between the number of students in classrooms – or classroom size – and the prevalence of bullying and victimization. Although it is often assumed that bullying occurs more frequently in larger classes, evidence of a positive relationship between class size and bullying or victimization is scarce in the literature (e.g., Khoury-Kassabri et al., 2004). Instead, these variables were generally found to be either unrelated (e.g., Thornberg et al., 2017) or negatively associated (e.g., Vervoort et al., 2010). The reasons for this negative association have, to our knowledge, never been investigated. In addition to testing whether the links between classroom size and bullying and victimization - both self-reported and peer-reported - would be negative in two different samples (from Austria and the Netherlands), our objective was to account for the higher rates of bullying and victimization often found in smaller classrooms by putting three explanations to the test. Across the two samples, all three hypothesized mechanisms received some support from the data, but none of them emerged as a clear, unequivocal reason for the negative association.

First, it is important to note that across the two samples, no evidence was found that bullying or victimization would occur less in smaller classrooms. In other words, there was no indication that belonging to a small classroom would have a protective effect against bullying. Consistent with our expectations, rates of peer-reported bullying and victimization were lower in larger classrooms. Classroom size was negatively associated with both self-reported and peer-reported bullying and victimization in the Dutch sample, but negatively associated with peer-reported measures only in the Austrian sample. Although the findings differed between the two samples, classroom size explained a higher proportion of variance in peer-reported than in self-reported bullying and victimization in each sample. These findings suggest partial support for our first hypothesis, which was that the negative effects of classroom size would only be due to a measurement effect, and therefore would be observed for peer-reported bullying and victimization, but not for self-reports. Our findings suggest that, consistent with our review of the literature, the type of measurement used may play a role in accounting for this negative link. This is likely related to a higher probability for students to receive high peer-reported scores in smaller networks. In that regard, it is noteworthy that classroom size was negatively associated with the other peer-reported measures used in the present study, namely perceived popularity and friendships. Nonetheless, the negative links found between classroom size and self-reports of bullying and victimization in the Dutch sample indicate that factors other than measurement must also be at play.

Although no evidence was found that the friendships of either victimized children or bullying perpetrators would be dependent on classroom size, our results did show a moderating effect of classroom size on the association between popularity and bullying in the Austrian sample, and between popularity and victimization in the Dutch sample. Consistent with Hypothesis 2, bullying perpetrators appear to be perceived as more popular in smaller compared to larger classrooms. In their social relationships, young bullying perpetrators tend to aim for control and influence, as suggested by positive associations between bullying and agentic goals (Caravita and Cillessen, 2012). Also, the most popular students in a classroom tend to be the most visible and dominant ones. Smaller networks should facilitate bullying perpetrators’ attempts at intimidating others and damaging their reputation, as well as maintaining their own position at the top of the social hierarchy. Larger classrooms should be more likely to be divided into multiple peer groups, making it easier for at least some students in the network to escape the influence of the ringleader bullies and their followers, thus decreasing their power relative to smaller networks. Being better rewarded with status in smaller classes should encourage bullying perpetrators to pursue their conduct, thus partly explaining why bullying may be more prevalent in smaller social environments. However, this finding should be interpreted with caution, as it was not found in the Dutch sample. Future research should examine whether the effects of classroom size on the popularity-bullying link depend on the type of aggression displayed by the bullying students. Some of them might use exclusively relational forms of aggression, such as rumor spreading and exclusion, that rely on the manipulation of the whole peer network more than physical aggression, which can occur in one-to-one bully-victim interactions.

Partial support was found for the proposition that the negative association between victimization and popularity would be stronger in smaller classrooms (Hypothesis 3). In smaller classes, differences in popularity between victimized children and non-victimized were larger than in larger classrooms. This finding is consistent with the idea that it may be more difficult for victims of bullying to have social power in smaller compared to larger classrooms, which in turn should promote higher victimization in smaller classrooms. Nevertheless, classroom size seemed to make a difference especially for the popularity of the students low on victimization. Therefore, this effect, which was observed in only one of the two samples, should also be interpreted cautiously.

Unfortunately, the lack of clear support across the two samples for any of the three hypotheses tested in the present study indicates that none of them stands out as a convincing explanation for the higher prevalence of bullying and victimization in smaller classrooms. This calls for further investigation of the factors accounting for this association. A factor that is likely to play a role is the tendency for certain schools to place children with disruptive behaviors in smaller classrooms to facilitate classroom management for teachers and make it easier for them to give attention to these children. However, no official record of these practices were available to the researchers, and for this reason they could not be controlled for in our analyses. There is also no evidence that these practices do occur or to what extent they occur. Moreover, children who display behaviors that are disruptive to teachers and school staff may not necessarily be the same children who are involved in bullying incidents with peers, either as perpetrator or target. However, it would be an important possibility to explore in future investigations.

One should also consider the possibility that some schools may have policies which require to place additional adult supervisors in large classrooms. In this case, the adult-child ratio might actually be lower in larger classrooms than in smaller classrooms, which could partly explain the negative relationship between class size and bullying. Unfortunately, no data was collected on the implementation of such practices.

### Limitations

This study focused on the effects of a classroom characteristic on the prevalence on individual behavior. However, the proportions of variance in bullying and victimization explained by differences between classrooms ranged from 4 to 15% across the two samples. These low numbers indicate that most of the variation in bullying and victimization is explained by individual characteristics. Therefore, even when the effect of classroom size on bullying is significant, this finding should not be interpreted as evidence that the number of students in the classroom plays a major role in bullying behavior. For self-reported measures in particular, classroom size explained less than 17% of the variation between classrooms in either bullying or victimization. Moreover, the significant quadratic effects found with the Dutch data indicate that there is a limit to the beneficial effects of larger classrooms for bullying problems, as these effects seem to disappear once classrooms reach a size of 29 students.

Further, we chose to examine the relationship between size of the peer network and bullying using the classroom as the unit of analysis. In the literature, however, school size was also investigated in relation to the prevalence of bullying and victimization, with mixed findings (see Luyten et al., 2014). The classroom seemed to be the most relevant unit for our analyses, as all children within a classroom generally know each other and are more likely to interact with each other than children of the same school. Moreover, peer nominations of social behavior and status are easier to collect within classrooms. Nevertheless, studies of adolescent cliques also found negative links between clique size and relational aggression (Pattiselanno et al., 2015). Therefore, future research may consider examining links between peer group size and bullying. Focusing on peer groups might even facilitate the investigation of the mechanisms through which the size of the peer network may promote or hinder bullying behavior.

Our cross-sectional analyses did not give any indication regarding decreases or increases in bullying and victimization across the school year in large versus small classrooms. They also did not allow us to determine whether the moderating effect of class size on the bullying-popularity relationship found in the Austrian sample was due to bullying perpetrators becoming more popular or to popular students increasing their bullying behavior in smaller classrooms. A better understanding of the role of classroom size in bullying behavior will require longitudinal investigations of these behaviors and of indicators of status or social adjustment that are relevant for explaining dynamics of bullying and victimization.

The effects of class size on bullying might be moderated by other factors, which were not considered in the present study. Research shows that teachers may play a role in preventing or maintaining bullying and victimization in their classrooms: Bullying rates are lower in classrooms where teachers report greater commitment to prevent bullying (Espelage et al., 2014), and victimization rates are higher in classrooms where teachers attribute bullying to factors outside of their control and feel less capable of handling bullying among students (Oldenburg et al., 2015). The possible adverse effects of smaller classrooms on bullying issues may therefore be mitigated by the conduct of teachers regarding these problems. It will be important for future studies examining if and why smaller class environments promote bullying, to test whether these effects are moderated by teachers’ handling of bullying cases or more generally, by their classroom management style.

Finally, it is possible that the differences in the results observed between the two samples are due to differences in the operationalization of the variables. For example, peer nominations of popularity were limited to five in the Austrian sample and unlimited in the Dutch sample, which means that the measurement error may have been higher and estimates less accurate in the Austrian sample (Gommans and Cillessen, 2015). Also, in the assessment of bullying in the Austrian sample, participants were not instructed to take into account power differential and repetition, which implies that it captured aggression more than bullying specifically. However, since the goal of our study was not to compare two samples or two countries, these differences in assessment do not invalidate our analyses or findings.

## Conclusion

In the field of education, the topic of class size has received considerable attention, primarily because class size reduction represents a convenient policy instrument (Hanushek, 1999). It is however, a controversial topic, as the extensive research conducted on the link between classroom size and academic achievement or social adjustment has not always yielded consistent findings (see Mishel and Rothstein, 2002). The present study aimed to clarify the relationship between bullying and classroom size by putting to the test explanations for the somewhat counter-intuitive finding of a higher prevalence of bullying problems in smaller classrooms. Our results provide further evidence that smaller classrooms have no protective effect against peer abuse. However, the reasons for the negative link between classroom size and bullying or victimization are not fully elucidated yet. Our study suggests that it is unlikely that a single mechanism is at play. Our findings should encourage researchers to consider the type of measurement used, as well as the possibility that bullying perpetrators might enjoy greater social power in smaller classes, when interpreting this negative association. Replications studies using longitudinal data and examining potential mediators and moderators of this association are needed.

## Data Availability

The datasets generated for this study are available on request to the corresponding author.

## Ethics Statement

Ethical review and approval was not required for the study on human participants in accordance with the local legislation and institutional requirements. Written informed consent to participate in this study was provided by the participants’ legal guardian/next of kin.

## Author Contributions

CG conceptualized the study, conducted the statistical analyses, and wrote the manuscript. TY assisted with the statistical analyses and provided feedback on the methodology. MV, DS, and CS provided feedback on the manuscript.

## Conflict of Interest Statement

The authors declare that the research was conducted in the absence of any commercial or financial relationships that could be construed as a potential conflict of interest.
